# Docosahexaenoic Acid (DHA) Inhibits FADS2 Expression in Astrocytes but Increases Survival of Neurons Co-cultured with DHA-enriched Astrocytes

**DOI:** 10.22088/IJMCM.BUMS.8.3.232

**Published:** 2019

**Authors:** Dorota Bewicz-Binkowska, Emilia Zgorzynska, Barbara Dziedzic, Anna Walczewska

**Affiliations:** *Department of Cell-to-Cell Communication, Medical University of Lodz, Poland*

**Keywords:** Docosahexaenoic acid, FADS2, astrocytes, neuroprotection

## Abstract

Docosahexaenoic acid (DHA), the most abundant n-3 polyunsaturated fatty acid (n-3PUFA) in the brain, has attracted great importance for a variety of neuronal functions such as signal transduction through plasma membranes, neuronal plasticity, and neuroprotection. Astrocytes that provide structural, functional, and metabolic support for neurons, express ∆6- desaturase encoded by *FADS2 *gene that can be, next to the plasma DHA pool, additional source of DHA in the brain. Furthermore, the genetic variations of *FADS* gene cluster has been found in children with developmental disorders, and are associated with cognitive functions. Since, the regulation of DHA biosynthesis in astrocytes remains poorly studied the aim of this study was to determine the effect of palmitic acid (PA), α-linolenic acid (ALA) or docosahexaenoic acid (DHA), on the transcription of *FADS2* gene in astrocytes and survival of neurons challenged with oxidative compounds after co-culture with astrocytes exposed to DHA. The lipid profile in cell membranes after incubation with fatty acids was determined by gas chromatography, and *FADS2* expression was analyzed using real-time PCR. The viability of neurons cocultured with PUFA-enriched astrocytes was investigated by flow cytometry after staining cells with annexin V-FITC and PI. The results showed that DHA suppressed (P <0.01), PA stimulated (P <0.01), while ALA did not change the *FADS2* gene expression after 24 h incubation of astrocytes with fatty acids. Although *FADS2* mRNA was down-regulated by DHA, its level in astrocytic membranes significantly increased (P <0.01). Astrocytes with DHA-enriched membrane phospholipids markedly enhanced neuronal resistance to cytotoxic compounds and neuronal survival. These results suggest that beneficial effects of supplementation with n-3 PUFA in Alzheimer disease and in psychiatric disorders is caused, in part, by increased efficacy of DHA-enriched astrocytes to protect neurons under adverse conditions in the brain.

The brain is highly enriched in docosahexaenoic acid (DHA 22:6n-3) which is of particular significance for the brain development and function (1). DHA esterified at sn-2 position of phospholipids in plasma membranes forms one third of the total fatty acids in the CNS (2). It has been shown that DHA presence in membranes affects the organization of lipid rafts and plays an important role in modulation of intracellular signaling and synaptic plasticity. Hence, a reduced DHA level in brain phospholipids is associated with a decline of cognitive functions in elderly, as well in patients with Alzheimer’s disease (3) and with psychiatric disorders (4, 5). The main source of DHA for the brain is uptake from esterified blood pool of DHA after dietary fat digestion (6). Moreover, DHA can be synthesized from essential -linolenic acid (ALA, 18:3n-3) in the liver through elongation and introducing *cis *double bonds into the hydrocarbon chain by desaturases (7). Delta 6-desaturase encoded by *FADS2 *gene is considered as the rate-limiting enzyme in the biosynthesis of long chain polyunsaturated fatty acids(LC-PUFAs). Astrocytic Δ6-desaturase expression has likewise been demonstrated (8) and association between *FADS2* single nucleotide polymorphisms and low LC-PUFA levels in erythrocyte membranes in patients with mild cognitive impairment was noted (9).

Astrocytes, the most abundant cells in the brain provide structural, functional and metabolic support for neurons. They are involved in maintaining ionic (10) and neurotransmitter homeostasis (11), as well participate in neuronal plasticity and interneuronal communication, as they express numerous receptors, metabolize amino acid neurotransmitters and release numerous gliotransmitters (12,13,14). Since biosynthesis of DHA in astrocytes remains poorly understood, the aim of this study was to determine DHA incorporation into plasma membranes and its effect on transcription of *FADS2* gene in astrocytes in comparison with other fatty acids, such as saturated palmitic acid (PA; C16:0) and the DHA precursor, ALA. Since cooperation between astrocytes and neurons, among others relies on supply of energy and metabolic substrates and protection against oxidative stress (15), we determined survival of neurons co-cultured with DHA-enriched astrocytes in response to cytotoxic agents.

## Materials and methods

**Primary astrocyte culture**

Astrocytes were prepared from the cerebral cortex of 1-2 days old rat pups using modified method of McCarthy and deVellis (16) and Albuqerque et al. (17). Briefly, cortices were dissected and dispersed into single cells by triturating in DMEM (Life Technologies, USA). The debris were removed by double filtration through Nitex 180 m, and then Nitex 30 m (Merck, Germany). The clear suspension was centrifuged (5 min, 800 rpm at room temperature), pellets were suspended in DMEM-F12/10% FBS medium, and cells seeded into 75 cm^2^ culture flasks (2 x10^5^ cells/cm^2^). Astrocytes were cultured in a 5% CO_2_ atmosphere at 37 °C, the medium was changed every two days until the cells were 70–80% confluent, then flasks were shaken to detach the oligodendrocytes. The monolayer cells were trypsinized, counted, plated into new 75 cm^2^ flasks and cultured to 80% confluence. The purity of astrocyte culture was 99%, as determined by glial fibrillary acidic protein (GFAP) (Life Technologies, USA) fluorescent immunocytochemistry. The study protocol was approved by the Ethical Committee for Animal Care.

**RNA preparation and real-time PCR**

To examine the effect of various types of fatty acids on *FADS2 *gene expression astrocytes were seeded in 6-well culture plates at 5x10^5^ cells/well and incubated with palmitic acid (PA), -linolenic acid (ALA) and docosahexaenoic acid (DHA) (Sigma Aldrich, USA) at concentration of 50 M for 24 h. Control cells were cultured in DMEM. Total RNA was isolated using the TRIZOL RNA reagent (Invitrogen, USA) *according to the manufacturer's instructions.* For the reverse transcription reaction pure RNA with a ratio A 260/280 of 1.9- 2.1 was used. First-strand cDNA was synthesized from 1 g of total RNA in a total 20 μl using Superscript III reverse/ImProm-IITM reverse transcriptase (Promega, USA), dNTPs and random hexamer primers (Invitrogen, USA) for 1 h at 50 °C. Then, RT products were analyzed in triplicate by real-time RT-PCR using an ABI Prism 7000 sequence detection system (Applied Biosystems, USA) with a Brilliant III SYBR QPCR kit (Agilent Technologies, USA) following the manufacturer’s protocol. PCR efficiency was validated with a standard curve. Primers used to detect the mRNA of *FADS2* and housekeeping gene glyceraldehyde-3-phosphate dehydrogenase (*GAPDH*) are presented in Table 1. The results were analyzed according to the 2^-ΔΔCt^ method. The mRNA level for each sample was normalized against *GAPDH* mRNA, and data are presented as relative quantification (RQ), i.e. fold change compared to the untreated control with RQ value of 1.

**Table1:** Primers used in the real-time PCR analysis

**Gene**	**Sequence ID**	**Primer sequences**	**Amplicon (bp)**
*FADS2*	HQ 909027.1	Forward:5’-GTTCTTCTTTCTCCTCCTGTCCC-3’	77
		Reverse:5’-CATTGCCGAAGTACGAGAGGAT-3’	
*GAPDH*	NM 017008.4	Forward: 5’-CATGGCCTTCCGTGTTCCTA-3’	105
		Reverse: 5’-CCTGCTTCACCACCTTCTTGA-3’	

**Membrane preparation and fatty acid determin-ation**

Astrocytes were plated at density of 8 x 10^6^ in Petri dishes and incubated with 30 M DHA for 24 h. Control cells were cultured in DMEM. Then cells were harvested, re-suspended in buffer (0.25 M sucrose, 1 mM EDTA, and 10 mM Tris-HCl, pH 7.4), and homogenized in a glass homogenizer with a Teflon pestle. Crude plasma membrane and mitochondria fractions were separated by differential centrifugation and then in sucrose density gradient. Lipids were extracted by standard Folch method, subsequently separated with the mobile phase: heptane-diisopropyl ether–acetic acid (60:40:3, v/v/v) and trans-methylated to fatty acid methyl esters (FAMEs) with boron trifluoride in methanol reagent under nitrogen atmosphere at 100 °C for 30 min. The FAMEs were analyzed by gas chromatography with a flame ionization detector (FID) on a Clarus 500 Gas Chromatograph (Perkin Elmer, USA). FAMEs were then separated on a capillary column coated with Varian CP-Sil88 stationary phase (50 m ×0.25 mm, ID 0.2 μm, Varian). Operating conditions were as follows: the split-splitless injector was used in split mode with a split ratio of 1:20. The injection volume of the sample was 2 μl. The injector and FID detector temperature was kept at 260 °C. The column temperature was programmed from 150 °C (2 min) to 230 °C (10 min) at 4 °C/min. The carrier gas was helium at a flow rate of 1 ml/min. FAMEs were identified by comparing their retention time with standards and quantitated using an internal standard method.

**Astrocyte-neuron co-culture**

Neurons for the astrocyte-neuron co-culture were obtained from the brain of rat pups at *postnatal* *day* *0–*1. After decapitation cerebral cortices were dissected and collected in cold DMEM with 10% FBS then purified from the meninges, chopped and digested at 37 C with papain (2 mg/ml) (Sigma Aldrich, USA) for 30 min and with DNase (2.5mg/ml) (Sigma Aldrich, USA) for 30 s. Following trituration through a fire-polished glass Pasteur pipette cell suspension was passed through a 70 m cell strainer and centrifuged (800 rpm, 5 min, room temperature). Obtained neurons were plated at a density of 5 x 10^4^ cells/well in poly-L-lysine coated 12-well culture plates and incubated in DMEM for 4 h. Then medium was changed to Neurobasal A enriched with 10% B27 supplement (Life Technologies, USA) and cells were allowed to settle for 5-10 days. ThinCert™ cell culture inserts (Greiner Bio-One, Austria) were utilized to obtain co-culture. Primary astrocytes were seeded in cell culture inserts with 1,0 m pores at density 1x10^5^ cell/insert for 24 h. Following 24 h incubation with 30 M DHA inserts with astrocytes were transferred to the wells containing neurons. Astrocytes and neurons were co-cultivated in DMEM supplemented with N2 and FBS for 24 h. Subsequently, inserts with astrocytes were removed and neurons were exposed to 40 M tBOOH and 0.45 mM H_2_O_2_ for 2 h.

**Flow cytometry**

The sensitivity of neurons co-cultured with DHA-enriched astrocytes to cytotoxic agents was evaluated by the quantification of viable, apoptotic and necrotic cells by Annexin V/propidium iodide (PI) (Life Technologies, USA) double staining and detection by flow cytometry. Neurons were harvested with trypsin-EDTA, washed with PBS and stained with Annexin V-FITC and PI for 15 min, at room temperature in the dark. The fluorescence intensity of 10,000 cells was measured in a FACSCanto II flow cytometer (Becton Dickinson, USA). The proportions of viable (Annexin−/PI−), apoptotic (Annexin V+/PI−), necrotic (Annexin V−/PI+) cells were analyzed using BD FACSDiva software. 

**Statistical analyzes**

The results are presented as means ± SEM. Statistical significance using GraphPad Prism 6.0 was determined by one-way ANOVA followed by the Newman-Keuls multiple comparison test. For analysis of neuron viability student’s t-test was applied. Statistical significance was considered at a P <0.05.

## Results

**Expression of** ***FADS2*** **gene in astrocytes**

**Fig. 1:**
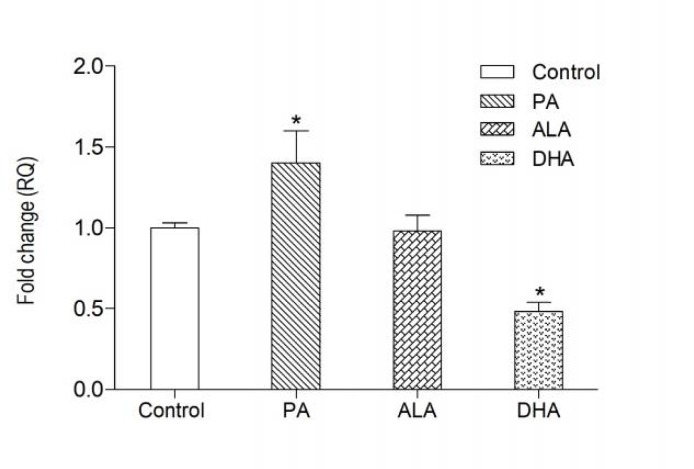
***FADS2*** **mRNA levels in astrocytes after incubation with palmitic acid (PA), ** **-linolenic acid (ALA) and docosahexaenoic acid (DHA)**. All fatty acids were used at 50  M concentration. Data are mean of fold change   SEM. * P < 0.01 compared to untreated astrocytes (control)

The transcription of *FADS2 *gene was significantly up-regulated in astrocytes supplemented with PA (P<0.01) and down-regulated by at least 50% in astrocytes incubated with DHA (P < 0.01) as compared to cells growing in the medium not supplemented with fatty acids. The level of *FADS2* mRNA in astrocytes incubated with ALA was not different from that in control cells (figure 1).

**Table 2:** Fatty acids composition of mitochondrial and plasma membranes of astrocytes

Fatty acids	% of total fatty acids				
	Mitochondrial membranes		Plasma membranes	
	Control	DHA-supplemented	Control	DHA-supplemented	
Saturated	59.67 0.79	56.06 0.77	48.13 0,57	47.63 1.1	
MUFA	25.15 0.19	21.10 0.14	29.44 0.32	23.92 0.42	
PUFA n-6	8.02 0.29	6.28 0.06	10.39 0.42	7.18 0.37	
C20:5n-3 (EPA)	0.65 0.03	0.45 0.05*	1.4 0.06	0.96 0.07*	
C22:6n-3 (DHA)	6.51 0.22	16.12 0.8**	10.65 0.11	20.33 0.41**	

Values are mean SEM. *P < 0.05; **P < 0.01 compared to control.

**Fig. 2:**
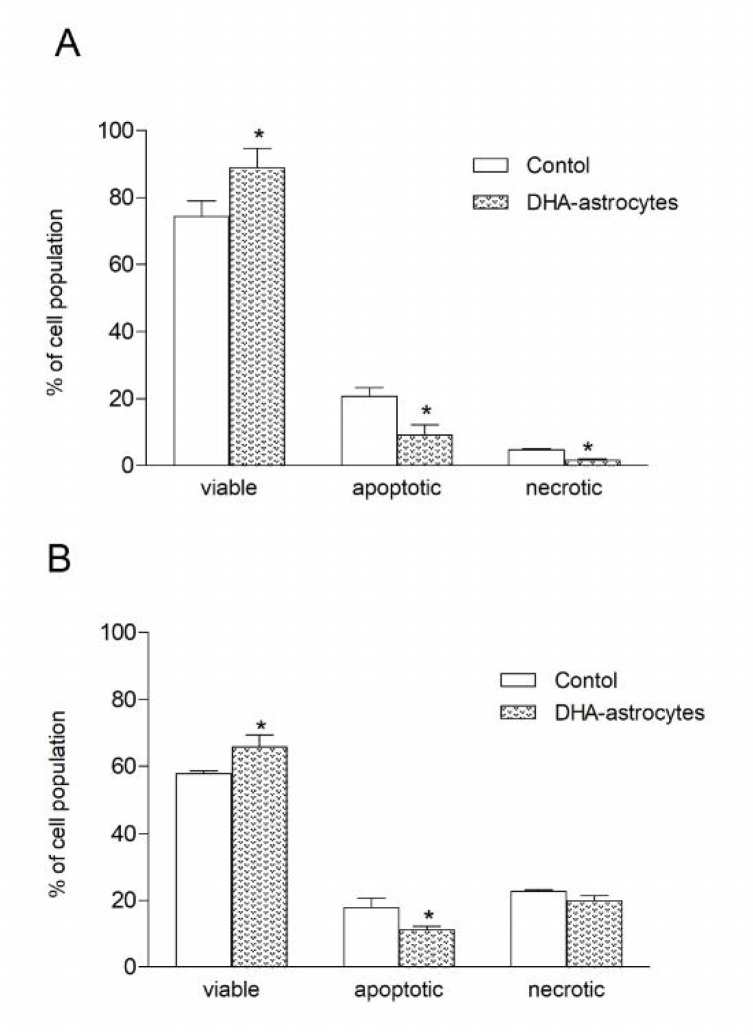
Survival **of neurons co-cultured with astrocytes preincubated with DHA.** Cells were challenged with tBOOH (A) or H_2_O_2 _(B); cells growing in DMEM were used as control. Data are mean   SEM, * P < 0.05 compared to control

**Incorporation of DHA into astrocyte membranes**

The fatty acid composition in mitochondrial and plasma membrane of astrocytes incubated with DHA is shown in Table 2. Incubation of astrocytes with DHA resulted in about 2.5-fold increase in the content of this fatty acid in mitochondrial as well as about 2-fold rise in plasma membranes compared to control cells. 

**Survival of neurons co-cultured with DHA-enriched astrocytes**

Figure 2 shows the quantification of viable, apoptotic and necrotic populations of neurons co-cultured for 24 h with DHA-enriched astrocytes compared to neurons co-cultured with astrocytes growing in standard medium without fatty acid (in figure depicted as control). Neurons co-cultured with astrocytes growing in DMEM after exposure to t-BOOH and H_2_O_2 _survived in 74% and 58%, respectively. Neurons co-culture with DHA-enriched astrocytes manifested a significant increase in percentage of viable cells after the treatment with t-BOOH and H_2_O_2_ to 89% and 66%, respectively.

## Discussion

Our results showed that DHA suppressed, PA stimulated, while ALA did not change the *FADS2* gene expression in astrocytes. Dietary study in animals demonstrated that a diet rich in fish oil decreased the activity of 6-desaturase in the liver microsomes (18). However, changes in the enzyme activity may not result from changes in the gene and protein expressions. In our previous study, we showed that the effect of fatty acids on *FADS2 *mRNA expression did not reflect the levels of FADS2 protein in astrocytes (19). It should be considered that there is a competition between specific fatty acids at the subsequent stages of their biosynthetic pathway. The experiments on cells transfected with *FADS2* gene demonstrated LC-PUFA synthesis inhibition in excess of PA by competing ALA and linoleic acids for 6-desaturase which introduces double bonds at the first and the last stage of LC-PUFA biosynthesis (20).

The mechanism involved in the regulation of 6-desaturase by fatty acids is studied mostly in the liver (6) and adipose tissue (21), but rarely in astrocytes. In mammals, transcription of *FADS2 *is regulated by sterol regulatory element-binding protein 1 (SREBP-1) and peroxisome proliferator-activated receptor (PPAR) that act reciprocally on regulation of fatty acid metabolism (22,23) and as the sensors of PUFA levels in cells. PUFAs suppress the transcription of the hepatic lipogenic genes by reducing *SREBP-1c* mRNA and SREBP-1 protein levels, as well the posttranslational proteolytic activation of SREBP-1c (24), while saturated and monounsaturated fatty acids do not have such effect (25). It has been demonstrated that dietary fish oil decreased the 6-desaturase mRNA and protein expressions, both in the liver and the brain (26), and the 6-desaturase promoter has the functional sequence that binds SREBP 1 during suppression by PUFA (27). Furthermore, the 6-desaturase expression was increased in mice overexpressing the nuclear form of *SREBP* (23). These findings indicate that DHA-reduced *FADS2* transcription in our study may result from *SREBP-1c* inhibition. Alternatively, DHA as an endogenous ligand of retinoid receptor (RXR) (28, 29), the obligatory member of the heterodimer RXR/ PPARα (30) may regulate the expression of *FADS2* gene (31).

In a previous study (15), we showed that incubation of astrocytes with DHA enhanced the expression of the anti-oxidative GSH-dependent enzymes via the Nrf2 pathway, that is in accordance with other results (32, 33). In the present work we demonstrated that astrocytes with DHA-enriched membrane phospholipids protect neurons against death caused by cytotoxic agents. Since astrocytes supply glutathione (GSH) to neurons (our own unpublished results, 34), the neuroprotective effect of DHA-enriched astrocytes likely results from increased GSH delivery and enhanced neurons resistance to cytotoxic compounds. This is in line with the study on postmitotic human dopaminergic neurons cocultured with human or murine astrocytes before exposure to proteotoxic and oxidative stress. Astrocytes prevented neuronal apoptosis, enhanced degradation of aggregated poly-ubiquitinated proteins and increased resilience of neurons to cellular stress via continuously released GSH (35). This study demonstrates that *FADS2* expression was down-regulated after incubation of astrocytes with DHA, however, the DHA levels increased in the astrocytic membranes. The astrocytes with DHA-enriched membrane phospholipids enhanced neuronal resistance to cytotoxic compounds although the synthesis of LC-PUFAs could be reduced in response to elevated DHA levels. These results suggest that beneficial effects of supplementation with n-3 PUFA in early stages of Alzheimer disease and in psychiatric disorders is caused, in part, by increased efficacy of DHA-enriched astrocytes to protect neurons under adverse conditions in the brain, such as neuroinflammation or oxidative stress during ischemia.
